# Juvenile idiopathic inflammatory myopathies: the value of magnetic resonance imaging in the detection of muscle involvement

**DOI:** 10.1590/S1516-31802000000200002

**Published:** 2000-03-02

**Authors:** Maria Odete Esteves Hilário, Hélio Yamashita, Daniela Lutti, Cláudio Len, Maria Teresa Terreri, Henrique Lederman

**Keywords:** Juvenile dermatomyositis, Juvenile systemic lupus erythematosus, Muscle involvement, Magnetic resonance imaging, Dermatomiosite, Lupus Eritematoso Sistêmico, Comprometimento muscular, Crianças, Ressonância Magnética

## Abstract

**CONTEXT:**

One of the major current challenges related to juvenile idiopathic inflammatory myopathy is the search for highly sensitive and specific non-invasive methods for diagnosis as well as for follow-up.

**OBJECTIVES:**

The aim of our study was to describe typical magnetic resonance imaging findings and to investigate the usefulness of this method in detecting active muscle disease in juvenile dermatomyositis and juvenile systemic lupus erythematosus patients.

**DESIGN:**

Transverse study, blinded assessment.

**SETTING:**

University referral unit (Pediatric Rheumatology section, Department of Pediatrics, Universidade Federal de São Paulo / Escola Paulista de Medicina).

**SAMPLE:**

Thirteen patients (9 girls) with dermatomyositis, as well as 13 patients (12 girls) with juvenile systemic lupus erythematosus and 10 normal children (5 girls), were enrolled in the study.

**MAIN MEASUREMENTS:**

Qualitative and quantitative analyses of gluteus maximus, quadriceps, adductors and flexors were performed and evaluated by two radiologists, blinded to all clinical information. Spin-echo in T1, DP, T2 and IR was used in all MRI images.

**RESULTS:**

The different muscle groups presented non-uniform involvement in the patients. The patients with dermatomyositis presented acute and chronic muscular alterations, while those with lupus presented only chronic myopathy, especially atrophy. In the dermatomyositis group, the major alterations were found in the gluteus and flexor regions (signal intensity and fat replacement). The signal intensity was increased in all acute myopathies.

**CONCLUSION:**

The qualitative and quantitative resonance analyses are useful in detecting clinically active disease in patients with dermatomyositis.

## resumo

**CONTEXTO:**

Um dos maiores desafios relacionados as miopatias inflamatórias idiopáticas juvenis está na procura de métodos sensíveis e específicos, porém não invasivos, para o seu diagnóstico e acompanhamento.

**OBJETIVO:**

Avaliar os achados da ressonância magnética e investigar a utilidade do método na detecção do comprometimento muscular em pacientes com dermatomiosite juvenil e lúpus eritematoso sistêmico juvenil.

**TIPO DE ESTUDO:**

Transversal, simples cego.

**LOCAL:**

Setor de Reumatologia Pediátrica do Departamento de Pediatria da Universidade Federal de São Paulo / Escola Paulista de Medicina.

**MÉTODOS:**

Fizeram parte do estudo 13 pacientes (9 meninas) com dermatomiosite juvenil, 13 pacientes (12 meninas) com lúpus eritematoso sistêmico juvenil e 10 crianças (5 meninas) normais como controles. Em todas as imagens da ressonância foi utilizado o spin-eco em T1, DP, T2 e IR e realizadas as análises qualitativa e quantitativa dos glúteos máximos, quadríceps, adutores e flexores. Os exames foram avaliados por dois radiologistas que não tiveram acesso a qualquer informação sobre os pacientes.

**RESULTADOS:**

Mostraram comprometimento não uniforme dos diferentes grupos musculares em todos os pacientes. Alterações agudas e crônicas foram observadas nos pacientes com dermatomiosite enquanto que naqueles com lúpus foram encontradas apenas alterações crônicas, especialmente atrofia. As alterações mais importantes (substituição gordurosa e aumento da intensidade de sinal) foram observadas nos glúteos e flexores dos pacientes com dermatomiosite. Aumento da intensidade de sinal esteve presente em todas as miosites agudas.

**CONCLUSÃO:**

A análise qualitativa e quantitativa da ressonância magnética é útil para a detecção do comprometimento muscular clinicamente ativo em pacientes com dermatomiosite.

## INTRODUCTION

Idiopathic inflammatory myopathy syndrome is constituted by a heterogeneous group of diseases, of unknown etiology, that have in common an inflammatory muscle process.

It is characterized by proximal muscular weakness, elevation of serum enzymes related mainly to striated musculature, electromyograph alterations, presence of mononuclear infiltrate in muscles and absence of recognizable etiology.^1^ This group of diseases presents a large range of histopathological findings, response to treatment and clinical evolution.

The principal representatives of idiopathic inflammatory myopathies are dermatomyositis, polymyositis and inclusion body myositis. Juvenile dermatomyositis is the most important during infancy, as it occurs most frequently and presents the most severe muscular inflammation. There are also myopathies associated with other connective tissue diseases, especially juvenile systemic lupus erythematosus, scleroderma and juvenile rheumatoid arthritis.

One of the major current challenges related to idiopathic inflammatory myopathies is the search for highly sensitive and specific non-invasive methods for diagnosis as well as for treatment follow-up. Among the image diagnosis methods, magnetic resonance imaging (MRI) has shown itself to be one of the best techniques for this purpose and for muscle skeleton disease follow-up.^2,3^

In 1987, Kaufman et al,^4^ in a study carried out with dermatomyositis patients, observed a correlation between inflammatory muscle activity and some parameters analyzed by resonance. In later studies, undertaken mainly with adult patients, the usefulness of this method in the diagnosis of acute and chronic inflammatory muscle diseases was observed.^5-7^ However, although idiopathic inflammatory myopathies in childhood present their own characteristics, studies using MRI for muscular evaluation are not common.^8-10^ This fact motivated us to study resonance alterations in patients with juvenile dermatomyositis and juvenile systemic lupus erythematosus, and to correlate the findings quantitatively and qualitatively with the presence of inflammatory activity.

## METHODS

The procedures that follow were in accordance with the ethical standards of the committee responsible for human experimentation and with the Helsinki Declaration of 1975, as revised in 1983.

### Design

Transverse study, blinded assessment.

### Setting

Academic referral unit (Pediatric Rheumatology section, Department of Pediatrics, Universidade Federal de São Paulo / Escola Paulista de Medicina).

### Sample

Twenty six patients divided into 2 groups were evaluated over a period of 2 years: 13 with dermatomyositis (9 girls) diagnosed according to the Bohan and Peter (1975) criteria,^11^ with ages ranging from 5.6 to 16.6 years (mean of 11.7 years); 13 patients with juvenile systemic lupus erythematosus (12 girls) according to the American College of Rheumatology criteria,^11^ with ages ranging from 9.3 to 16.7 years (mean 13.6). The mean disease duration was 4.5 years (range 1.5 to 13.6) for dermatomyositis and 3.7 years (range 0.4 to 8.6) for lupus patients. We also calculated the mean daily corticosteroid dose for each patient during the last 6 months and correlated it with the MRI findings. The control group consisted of 10 healthy children and adolescents (5 girls), with ages ranging from 5.0 to 16.0 years (mean of 11.6 years), free from any disease.

### Diagnostic Procedures

*Clinical Evaluation.* The characterization of inflammatory activity in the dermatomyositis group was based on the clinical parameters of muscular strength and the presence of altered muscle enzymes, particularly the creatine kinase, aspartate aminotransferase, aldolase and lactic dehydrogenase. For lupus patients, the Systemic Lupus Erythematosus Disease Activity Index (SLEDAI) and the following scores were used:^13^ mild disease activity (score 1 to 9), moderate (score 10 to 19) and severe (score ≥ 20) disease activity. All parents gave informed consent for the study, which had the prior approval of the local ethics committee.

*Magnetic Resonance Imaging.* To carry out the MRI, the equipment used was a Philips 1.5-tesla Gyroscan S15, release 5.6. We performed T1-weighted and T2-weighted images with spin-echo (SE) sequences and fat suppression technique, with an inversion recovery (IR) sequence. The T1-weighted images were acquired in the coronal and axial planes: SE, TR/TE of 500-640/ 20ms, 2 to 4 acquisitions, 180 × 256 matrix, 10/10 mm thickness/increase and field of view (FOV) of 350 mm on the axial and 500 mm on the coronal. The T2 images were acquired in the axial plane: SE, TR/TE of 2500/90 ms, 1 acquisition, 204 × 256 matrix, 10/10 mm thickness/increase and FOV of 300 mm. The IR images were acquired in the axial plane: TR/TE/TI of 1900-2000/ 20/160 ms, 2 acquisitions, 180 × 256 matrix, 10/10 mm and FOV of 300 mm. Each patient and control was submitted to an MRI exam (total of 36). T1-weighted, T2-weighted and IR images were acquired. All images were obtained with the center in the mid-thigh.

### Main Measurements

The muscle groups studied were: gluteus maximus, quadriceps, adductors and flexors. The exams were evaluated by 2 radiologists who carried out qualitative and quantitative analysis of the 4 muscle groups by consensus, with no knowledge of the patient history. For the qualitative analysis in T2-weighted, T1-weighted and IR, 6 types of findings were standardized and given points in accordance with Table 1. The first 4 (signal intensity, chemical shift, perimuscular edema and signal intensity of subcutaneous fat) are findings of acute alterations and were observed on T2-weighted images. The other 2 parameters were observed on T1-weighted and IR images. With this method the maximum possible score obtainable on evaluation of each patient was 21 points. A lower score indicated lesser muscular problems. The analysis of muscular atrophy was made based on the relationship between the thigh muscle diameter and the total thigh diameter.

**Table 1:** Qualitative Analysis - score of each parameter analyzed

Findings	Score	
	0	1
↑ muscular signal intensity	≤ 50%	> 50%
↑ biochemical shift	No	Yes
Perimuscular edema	No	Yes
↑ signal intensity of subcutaneous fat	No	Yes
Muscular atrophy	≥ 0.75	< 0.75
Muscular fat replacement	≤ 50%	> 50%

The quantitative analysis was undertaken in T2-weighted images and the signal intensity of each individual group of muscles was obtained using a region of interest (ROI) of 0.5 cm at the midpoint of the thigh and lower third of the hip. The relationship between intensities of the muscle/fat signal was calculated by means of dividing the signal intensity measurements of the muscle groups by the fat measurements. For each muscle group, the mean of the 3 measurements obtained in the region with the strongest signal of the section being studied was used.

### Statistical Methods

The following non-parametric tests were used to analyze the results: Kruskal-Wallis test, exact Fisher test, Mann-Whitney test and the Friedman ranked variance analysis test. In all the tests, the level for the rejection of the null hypothesis was fixed at 5% (*P* < 0.05).

## RESULTS

Muscular weakness was observed in 9/13 patients (68%) with dermatomyositis, of whom 6 (46%) presented elevated muscular enzymes. The neck flexor muscles were the most affected. Creatine kinase was the most frequently altered enzyme (83%). Disease activity was characterized in 46% of patients.

We did not observe any clinical muscle impairment or muscular enzyme elevation in patients with lupus. As regards SLEDAI, 4 patients presented mild disease activity, 5 moderate and 4 severe disease activity.

### Magnetic Resonance Imaging

**Figure 1:**
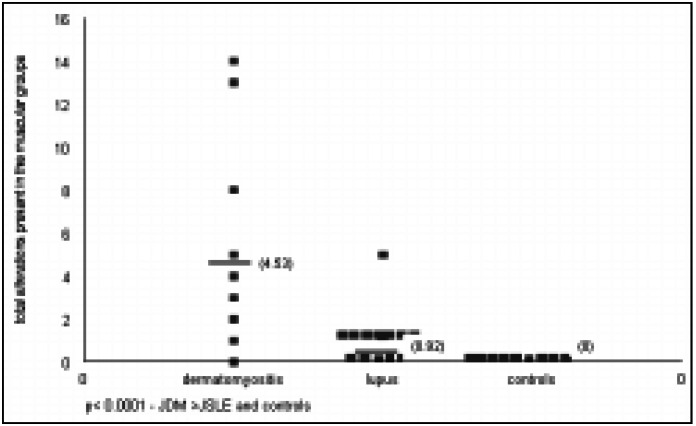
Patients with juvenile dermatomyositis, juvenile systemic lupus erythematosus and controls, according to the total alterations present in the muscle groups at qualitative analysis.

The frequency of alterations observed in the qualitative analysis was significantly higher in patients with dermatomyositis when compared to the lupus patients and controls (Figure 1). Among the variables studied, the muscle signal intensity and the chemical shift presented the most alterations, although there was no significant statistical difference (Table 2). The statistical analysis showed a significant association (*P* < 0.05) between the presence of acute findings and the dermatomyositis group. One interesting observation was the high frequency of chronic alterations in patients with lupus instead of acute alterations.

**Table 2:** Patients with dermatomyositis, according to the number of muscle groups affected in the qualitative analysis

Patient	↑muscular signal intensity	↑biochemical shift	perimuscular edema	↑subcutaneous fat signal intensity	muscular atrophy	muscular fat replacement
**1**	0	2	0	0	1	1
**2**	2	2	1	2	1	0
**3**	0	0	1	0	0	0
**4**	0	1	1	1	1	0
**5**	1	1	0	0	1	2
**6**	0	0	1	0	1	1
**7**	0	1	0	0	1	0
**8**	4	4	1	0	1	4
**9**	0	0	0	0	1	0
**10**	0	0	0	0	0	0
**11**	0	0	0	0	1	0
**12**	3	4	4	1	1	0
**13**	1	0	0	2	0	0
**Mean**	**0.84**	**1.15**	**0.69**	**0.46**	**0.69**	**0.61**

**Figure 2:**
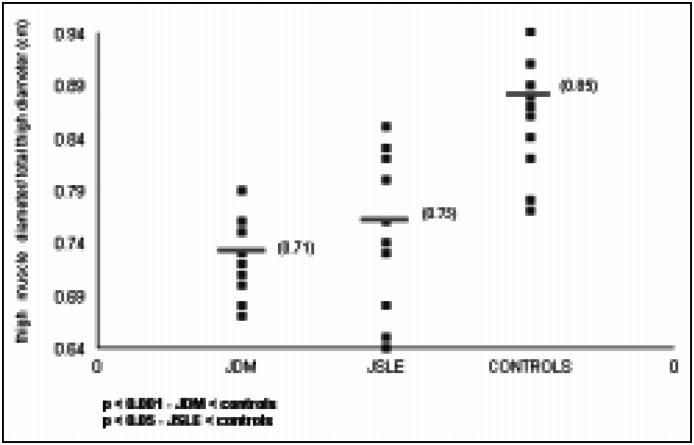
Patients with JDM, JSLE and controls, according to thigh muscle diameter/total thigh diameter (muscle atrophy).

In relation to muscular atrophy, the greatest involvement was observed in patients with dermatomyositis: this was less pronounced in patients with lupus, and not present in the controls (Figure 2). Quantitative analysis showed greater involvement of gluteus maximus and quadriceps in dermatomyositis patients as compared to controls.

**Figure 3:**
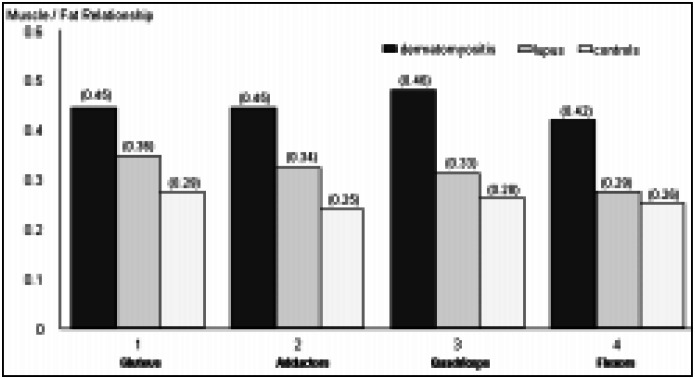
Relationship between the intensity of the muscle/fat signal in juvenile dermatomyositis, juvenile systemic lupus erythematosus and control groups.

In Figure 3 the muscle/fat relationship mean can be observed for muscle groups in patients with dermatomyositis, lupus and controls. The correlation between MRI, muscular weakness and elevation of muscle enzymes was determined only for patients with dermatomyositis, as the alterations were not observed in the lupus group. Regarding muscular weakness, a statistically significant association with the acute alterations was found and a tendency towards an association with the chronic alterations of the qualitative analysis was found. The patients with dermatomyositis and more accentuated muscular weakness presented a higher muscle/fat relationship and therefore greater involvement in the quantitative analysis (*P* = 0.008).

The increase in muscle enzymes also showed a significant correlation with the acute changes observed in the MRI qualitative analysis (P=0.004). Qualitative analysis variables only presented a statistically significant correlation with the biochemical shift analysis (*P* < 0.05).

Quantitative analysis showed a statistically significant correlation between higher values in the muscle/fat relationship and the raising of muscle enzymes (*P* = 0.015). In the dermatomyositis group, the mean daily corticosteroid dose was 0.59 mg (0 to 1.2 mg/kg/day) and higher doses only presented a correlation with the muscle/fat relationship (quantitative analysis). In the lupus group we did not observe a correlation between the mean corticosteroid dose (0.47 mg/kg/day) and the MRI findings.

## DISCUSSION

We evaluated 13 patients with juvenile dermatomyositis, 13 patients with juvenile systemic lupus erythematosus and 10 normal children and adolescents, using MRI exams with the spin-echo technique, with T1-weighted, T2-weighted and IR (fat suppression) images. The aim of this study was to correlate the clinical and laboratory alterations with the MRI findings. The study was performed with 2 types of analysis, the qualitative and the quantitative.

Characterization of disease activity in our dermatomyositis group was based on changes in muscular strength associated with the presence of elevated muscle enzymes, which will be discussed. As we know, muscle enzymes do not correlate well with muscular strength, rashes or muscle biopsy in some patients.^14,15^ On the other hand muscle weakness, in chronic patients, may just reflect muscular atrophy and fibrosis related to continuous or chronic-recurrent inflammatory disease. In spite of all these considerations, we decided to employ this criterion, since it is widely used in the literature.^4,6,16^

Disease activity was diagnosed in 46% of our patients with dermatomyositis. All lupus patients presented disease activity ranging from mild to severe, which was expected, as the group was heterogeneous in terms of disease duration and type of involve- ment.^17,18^ We did not observe clinical muscle involvement (muscle weakness) in these patients, as was described by other authors.^19,20^

Different to most studies published in the literature, which in general use 4 MRI parameters, we analyzed 6 types of findings for qualitative analysis: increase in muscle signal intensity, increase of chemical shift, perimuscular edema, increase in the intensity of the subcutaneous fat signal, muscular atrophy and muscle fat replacement. The frequency of the 5 qualitative MRI analysis findings (with the exception of atrophy) in the dermatomyositis group had no significant statistical difference, which may indicate that all the variables could have the same degree of importance. However, we must take into account that our patients were at different stages of activity. "Increase in the signal intensity" was able to detect patients with greater disease activity, presenting 80% sensibility and 75% specificity. These results are similar to those of Fujino et al,^19^ who observed an alteration in signal intensity in 7 of 8 patients with active dermatomyositis. The "chemical shift" detected alterations in 7 patients, 6 of whom with disease activity, thereby showing a sensitivity of 85.7% and a specificity of 83.3% in the detection of disease activity.

Muscular atrophy and fat replacement are qualitative analysis findings associated with chronic muscle processes. The cause of muscular atrophy in dermatomyositis is multifactorial and may be due to chronic inflammation with important muscular impairment, reduction of physical activity during disease activity and long term corticotherapy with elevated doses.^20^ It is probable that these 3 factors contributed to the presence of muscular atrophy in 84% (11/13) of the patients with dermatomyositis. The remaining patients were not active and were using low doses of corticosteroids.

Due to the small number of findings in patients with lupus, we did not carry out a statistical analysis. Nevertheless, it is interesting to note that 41% of the total qualitative analysis findings occurred in one patient, who at the time of the exam presented reactivation of the disease, with intense general manifestations and moderate disease activity (SLEDAI), although with no alterations in muscle enzymes or muscular strength. Chronic alterations predominated in lupus patients, represented mainly by muscular atrophy. This finding could be explained by some factors, such as the reduction in physical activity, prolonged corticotherapy with high doses, and perhaps the existence of a subclinical myopathy.

In summary, 92.3% (12/13) with dermatomyositis presented some degree of involvement in the qualitative resonance analysis, whereas the enzyme alteration was observed in 6 (46%). In the study of Park et al,^21^ the qualitative analysis alterations were present in 81.8% of patients, whereas the enzyme alterations amounted to 27%. These figures suggest that resonance is capable of detecting muscle involvement in patients with no clinical or enzyme alterations, perhaps by detecting subclinical disease activity and muscle scarring involvement. Some studies of patients with disease activity found resonance alterations in 100% of cases.^16,22^

Quantitative analysis is in general correlated with acute alterations in muscle involvement or active disease.^23^ In our study we observed significant differences among dermatomyositis, lupus and controls in 2 muscular groups (gluteus maximus and quadriceps). In the study of Hernandez et al,^22^ the muscle/ fat relationship mean for 4 muscle groups in 4 children was evaluated at onset and 6 months after the start of treatment. These authors observed significant differences between the muscle/fat relationship mean for the gluteus maximus, adductors and quadriceps of patients with dermatomyositis. The difference between our findings and those of Hernandez et al,^22^ may have occurred because of heterogeneous muscle involvement, different degrees of disease activity and the longer disease duration observed in our patients. In our study, the muscle/fat relationship mean varied between 0.42 and 0.46. In the study of Hernandez et al,^22^ the mean values at the time of diagnosis were between 0.76 and 1.17 in the 4 muscle groups. After 6 months of treatment they observed a reduction in these relationships (0.41 to 0.61). This demonstrates that the muscle/fat relationship (quantitative analysis) is related to acute processes and that there is a tendency to normalization of this relationship during treatment.

## CONCLUSIONS

Magnetic resonance imaging is a useful method in the detection of muscular involvement and in the follow-up of patients with idiopathic inflammatory myopathies. It can be used in diverse ways because it is able to differentiate normal from pathologic musculature, to define acute and chronic muscle alterations and to detect and rank muscle involvement in relation to disease activity. In addition, it is a noninvasive, radiation free, painless examination that can be used repeatedly.
